# Illicit Stimulant Use in Humans Is Associated with a Long-Term Increase in Tremor

**DOI:** 10.1371/journal.pone.0052025

**Published:** 2012-12-18

**Authors:** Stanley C. Flavel, Jenna D. Koch, Jason M. White, Gabrielle Todd

**Affiliations:** 1 School of Pharmacy and Medical Sciences, University of South Australia, Adelaide, South Australia, Australia; 2 Sansom Institute, University of South Australia, Adelaide, South Australia, Australia; University of Toronto, Canada

## Abstract

Use of illicit stimulants such as methamphetamine, cocaine, and ecstasy is a significant health problem. The United Nations Office on Drugs and Crime estimates that 14–57 million people use stimulants each year. Chronic use of illicit stimulants can cause neurotoxicity in animals and humans but the long-term functional consequences are not well understood. Stimulant users self-report problems with tremor whilst abstinent. Thus, the aim of the current study was to investigate the long-term effect of stimulant use on human tremor during rest and movement. We hypothesized that individuals with a history of stimulant use would exhibit abnormally large tremor during rest and movement. Tremor was assessed in abstinent ecstasy users (n = 9; 22±3 yrs) and abstinent users of amphetamine-like drugs (n = 7; 33±9 yrs) and in two control groups: non-drug users (n = 23; 27±8 yrs) and cannabis users (n = 12; 24±7 yrs). Tremor was measured with an accelerometer attached to the index finger at rest (30 s) and during flexion and extension of the index finger (30 s). Acceleration traces were analyzed with fast-Fourier transform. During movement, tremor amplitude was significantly greater in ecstasy users than in non-drug users (frequency range 3.9–13.3 Hz; P<0.05), but was unaffected in cannabis users or users of amphetamine-like drugs. The peak frequency of tremor did not significantly differ between groups nor did resting tremor. In conclusion, abstinent ecstasy users exhibit an abnormally large tremor during movement. Further work is required to determine if the abnormality translates to increased risk of movement disorders in this population.

## Introduction

Individuals that use illicit stimulants such as methamphetamine, ecstasy, and cocaine report experiencing an abnormally large tremor whilst abstinent [1]. The term ‘tremor’ describes involuntary, rapid, and rhythmic oscillation of a body part. It can occur anywhere in the body, but is most commonly observed in the hands. Tremor occurs at a frequency of 8–12 Hz in healthy people and is more evident during movement than relaxation [2].

There has only been one study that has objectively measured tremor in abstinent stimulant users. Objective measurement of tremor involves the use of an accelerometer and spectral analysis of the acceleration trace. Use of this methodology suggests that abstinent cocaine dependent individuals exhibit an increased resting tremor that occurs at an abnormally low frequency [3]. However, the study contained several methodological flaws that limit interpretation of the results and the study did not quantify lifetime stimulant use or use of other types of illicit drugs. The latter is important because poly-drug use is common in stimulant users [4], [5]. For example, 70% of amphetamine and methamphetamine users in Australia currently use cannabis and 60% had used ecstasy in the past 12 months [6]. Stimulant use is also associated with higher consumption of alcohol and tobacco [7].

The aim of the current study was to investigate the long-term effect of illicit stimulant use on tremor at rest and during movement. We hypothesized that individuals with a history of illicit stimulant use would exhibit abnormal tremor at rest and during movement. Our hypothesis is based on self-reports of abnormal tremor in ecstasy users [1], prior work in abstinent cocaine dependent individuals [3], and a small number of studies that suggest subtle and overt movement dysfunction in stimulant users. For example, poor performance on timed gait and grooved pegboard tasks has been reported in abstinent methamphetamine [8], [9] and ecstasy users [10]. Furthermore, new diagnosed cases of dystonia, tic disorders, and choreiform syndrome have been attributed to cocaine use [11], [12], [13] and exacerbation of symptoms in pre-existing movement disorders has been noted with cocaine use in Tourette syndrome, essential tremor, tardive dystonia, and idiopathic dystonia [11], [14], [15], [16], [17]. Abnormal tremor could be a marker for abnormalities in motor circuitry.

## Materials and Methods

### Ethics Statement

The study was performed at the University of South Australia in Adelaide, Australia. All experimental procedures were approved by the Human Research Ethics Committee at the University of South Australia and Drug and Alcohol Services South Australia. Experimental procedures were conducted according to The Code of Ethics of the World Medical Association (Declaration of Helsinki) printed in the British Medical Journal (18^th^ July 1964). Written informed consent was obtained prior to participation.

### Experimental Protocol

Tremor was investigated in 51 healthy adults. Four groups of subjects were investigated: 9 subjects that had predominantly used ecstasy (termed ‘ecstasy group’), 7 subjects that had predominantly used amphetamine-like stimulants (termed ‘amphetamine group’), 12 cannabis users, and 23 non-drug users. The inclusion and exclusion criteria for the ecstasy group was use of ecstasy on ≥5 occasions and use of amphetamine-like stimulants on ≤5 occasions. Inclusion and exclusion criteria for the amphetamine group was use of amphetamine-like stimulants (i.e. amphetamine, methamphetamine, cocaine, and/or illicit use of dexamphetamine or methylphenidate) on ≥5 occasions and use of ecstasy on ≤5 occasions. Inclusion and exclusion criteria for the cannabis group were use of cannabis on ≥5 occasions but no history of stimulant use. The cannabis group was included because cannabis use is common among stimulant users. Inclusion and exclusion criteria for the control group were cannabis use on less than 1 occasion and no other history of illicit drug use (alcohol and tobacco use was permitted). Volunteers were primarily recruited via community advertisement (2 volunteers were recruited via a rehabilitation program). A total of 47 stimulant users were screened to identify subjects that fit the inclusion and exclusion criteria for the ecstasy (n = 9) and amphetamine (n = 6) groups.

Subjects underwent a series of screening tests prior to participating in the study. Subjects were asked to complete a brief medical history questionnaire [18] and provide a urine sample for routine drug screening (PSCupA-6MBAU, US Diagnostics Inc., Huntsville, Alabama, USA). Urine data is missing for 3 subjects (1 control subject, 1 amphetamine subject, and 1 cannabis subject) due to mislabeling of samples, although both drug users reported complete drug abstinence for 6 and 12 yrs, respectively. All subjects were then asked to complete a drug history questionnaire to document use of prescription, non-prescription, and complementary medicines within the last month and lifetime use of alcohol, tobacco, and illicit drugs. The questionnaire listed 20 illicit drugs and requested information on other illicit drugs not listed. Items on the questionnaire included age of first use, age of regular use, duration of use, frequency of use (current and lifetime), number of times used in the last year, average dose (current and lifetime), frequency of high dose use, time since last use, number of drug overdoses, and treatment for drug dependency. The final screening test involved a neuropsychological assessment of memory and cognition. Four cognitive domains were assessed. New learning was assessed with Logical Memory I and II [19], executive functioning was assessed with Verbal Trails and Verbal Fluency [20], [21], working memory was assessed with Digit Span backwards [22], and attention was assessed with Digit Span forwards [22]. Performance in each test was compared to published normative data matched for age and years of education. Symptoms of depression (over the past 2 weeks) were also assessed with a 21 item self-report rating scale (Beck Depression Inventory-II) [23] and speed of information processing was assessed with an inspection time test. The inspection time test involved presentation of two parallel lines on a computer screen and subjects were asked to indicate which of the two lines was shorter [24]. The minimum exposure time required to accurately determine the shorter line was recorded. The inspection time test is a measure of speed and efficiency of information processing and does not assess reaction time.

Exclusion criteria included a) history of neurological damage and/or neurological illness diagnosed by a clinician prior to illicit drug use, b) use of prescription medications that primarily act on the central nervous system in the last month (e.g. antipsychotics or antidepressants), c) use of medications (e.g. β-adrenoceptor antagonists) that are known to affect tremor [25], d) history of illicit opioid use on more than 3 occasions, and d) positive drug test for amphetamine, methamphetamine, MDMA (3,4-methylenedioxymethamphetamine or ‘ecstasy’), cocaine, opioids, and/or benzodiazepines. Subjects who tested positive for cannabis were allowed to participate if use was greater than 12 hours prior to the experiment. This exemption was due to the metabolite of the main active ingredient of cannabis (tetrahydrocannabinol) remaining in the body for up to 90 days after last use. Subjects were also excluded if poor performance was observed on 3 or more of the cognitive domains tested. Poor performance was defined as greater than 2 standard deviations below the mean of published normative data for digit span [26], verbal fluency [27], and logical memory I and II [28] and performance greater than 2 standard deviations above the mean for verbal trails [29].

**Figure 1:**
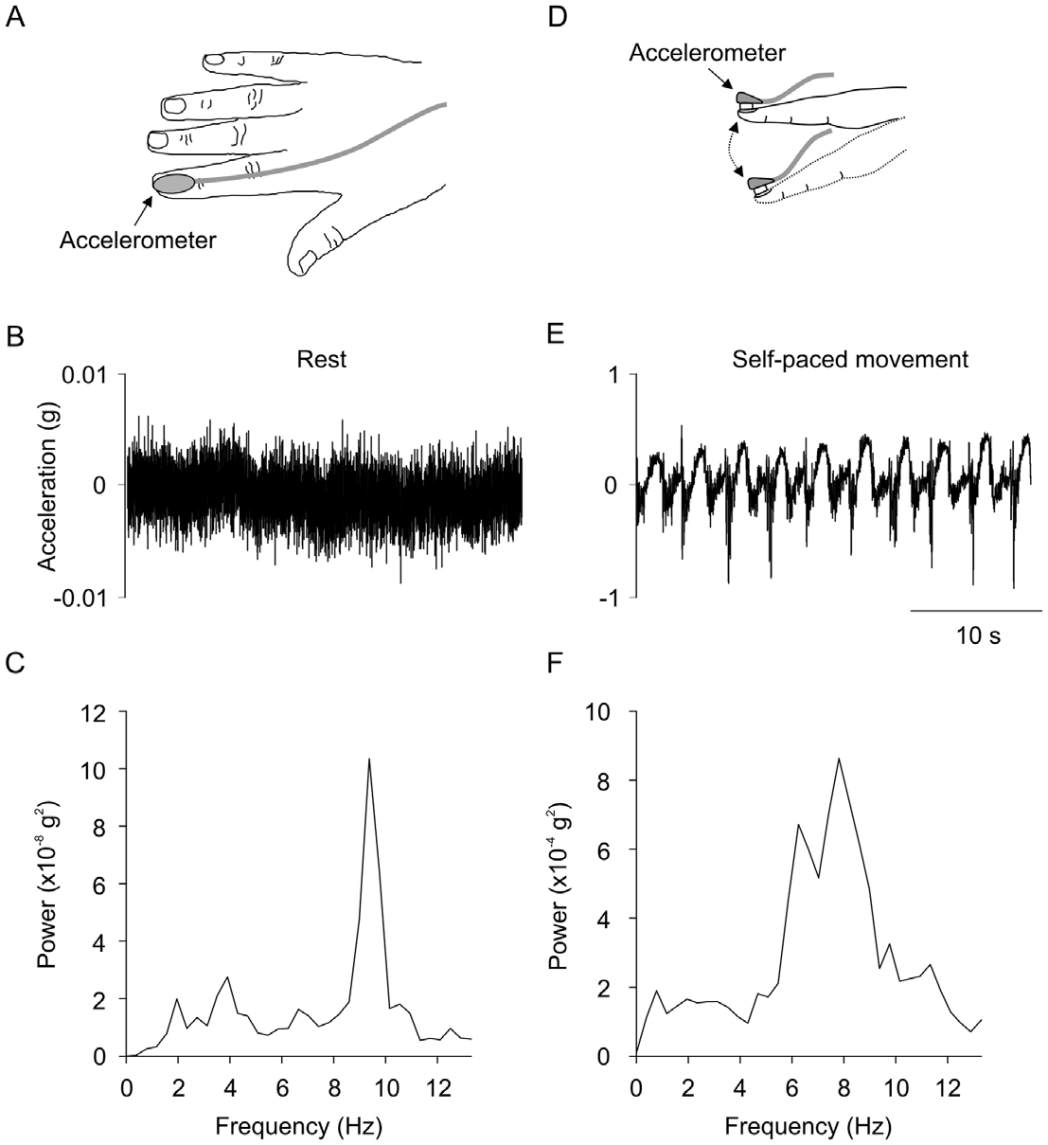
Experimental apparatus and acceleration traces from one subject. A) Experimental apparatus. B) Raw acceleration trace during relaxation. C) Result of the fast Fourier transform during relaxation. D) Flexion and extension of the index finger with the accelerometer attached. E) Raw acceleration trace during self-paced movement. F) Result of the fast Fourier transform during self-paced movement.

After completion of the screening tests, a small accelerometer (dimensions 5 mm x 5 mm x 2 mm; Precision ±2 g Dual Axis, PWM Output Accelerometer, ADXL212, Analog Devices, Norwood, Massachusetts; see Fig. 1A) was attached to the fingernail of the dominant index finger. The accelerometer enabled detection of very small perturbations in the vertical plane (i.e. finger flexion/extension) with respect to the acceleration due to gravity (g). Acceleration was sampled (400 Hz) and filtered (low-pass 100 Hz) with a data acquisition system (CED 1902 and Power 1401 with Spike2 software, Cambridge Electronic Design, Cambridge, USA). Tremor was measured at rest and during two flexion and extension tasks of the index finger (Fig. 1B) whilst blindfolded. Resting tremor was measured with the subject’s hand resting on a table (30 s duration). Subjects were then asked to raise their hand off the table for measurement of tremor during movement. The hand was positioned comfortably in front of the subject with the shoulder and elbow flexed and the wrist pronated. Subjects were asked to flex and extend their index finger (2 s duty-cycle) in a smooth manner for 30 s (termed ‘internally-paced movement’). Standardized verbal and visual instructions were given to each subject. Subjects then repeated the movement task with an auditory cue generated by a metronome (termed ‘auditory-paced movement’). The auditory cue was delivered at 1 Hz and signaled the turning point between flexion and extension (i.e. 2 s duty-cycle). Two movement conditions were investigated because symptom severity can differ between internally- and externally-paced movements in Parkinson’s disease patients [30].

### Data analysis

Data is presented as mean ± standard deviation in the text and mean ± standard error of the mean are shown in figures. Tremor analysis involved removal of the DC offset (time constant 0.25 s) from the raw acceleration trace followed by fast Fourier transform (FFT; size 1024, resolution 0.4 Hz, Hanning window) to construct a power spectral density. A separate FFT was performed for each condition. Data in the 0–3.9 Hz range was ignored because it comprised voluntary finger movement. The frequency range of interest was 3.9–13.3 Hz because it encompassed physiological tremor (8–12 Hz) and pathological tremor (e.g. 3–6 Hz in Parkinson’s disease) [31]. For each subject, mean and peak power was calculated for 3.9–6.6 Hz and 7.4–13.3 Hz. Mean and peak power are an index of tremor amplitude in the specified frequency range. The frequency corresponding to the peak power was also measured.

In the text, group data are presented as the mean ± standard deviation (SD), whereas in figures, mean ± standard error of the mean are shown. Subject characteristics (age, height, weight, education, depression score, speed of information processing), use of alcohol and tobacco, and resting tremor parameters were analysed with one-way analysis of variance (ANOVA) for comparison of group (control, ecstasy, amphetamine, cannabis). Duration of abstinence from cannabis was analysed with one-way ANOVA for comparison of groups (ecstasy, amphetamine, cannabis). Non-parametric data were transformed to ranks and repeated measures ANOVA on ranks were performed. Post-hoc discrimination between means was made with Student-Newman Keuls procedure (SigmaPlot 11.0, Systat Software Inc, Chicago, USA). Duration of abstinence from stimulants was analysed with unpaired Student’s t-test. For tremor during movement, group data was analysed with two-way repeated measures ANOVA for comparison of group (control, ecstasy, amphetamine, cannabis; between-subject factor) and movement condition (self-paced, auditory-paced; within-subject factor). Post-hoc analysis involved Dunnett t-tests for comparison of drug groups to the non-drug control group (IBM SPSS Statistics 20, IBM, Armonk, New York, USA). Pearson Product Moment correlation or Spearman Rank Order correlation was used to investigate the relationship between drug history parameters (lifetime ecstasy use (total number of occasions), duration of abstinence from stimulants, age of first ecstasy use, duration of ecstasy use) and tremor in the ecstasy group (SigmaPlot 11.0, Systat Software Inc, Chicago, USA). Statistical significance was set at P≤0.05.

## Results

### Subject Characteristics

Subject characteristics for each group are provided in Table 1. Groups were well matched for age, height, weight, handedness, years of education, and speed of information processing (i.e. inspection time). All subjects passed neuropsychological screening, but the score on the Beck Depression Inventory-II significantly differed between groups (F_3,47_ = 6.348, P = 0.001). As expected, symptoms of depression were more evident in the drug using groups (P<0.023) than in the control group. However, the depression score did not significantly differ between ecstasy, amphetamine, and cannabis groups. Two subjects in the ecstasy group had received a formal diagnosis of depression. The date of diagnosis occurred 2 and 10 yrs after commencement of illicit drug use, respectively, and subjects were not currently being treated with antidepressants.

**Table 1:** Subject characteristics for the control, stimulant, and cannabis groups.

	Control	Ecstasy	Amphetamine	Cannabis
Age (yrs)	27±8	22±3	33±9	24±7
Gender	11 M, 14 F	6 M, 3 F	5 M, 2 F	8 M, 4 F
Weight (kg)	66±12	69±12	80±22	78±20
Height (cm)	171±10	176±8	176±11	172±8
Handedness	22 right, 1 left	9 right, 0 left	7 right, 0 left	9 right, 3 left
Education (yrs)	15±2	16±2	16±4	15±2
BDI-II score	4±5	13±6*	11±7*	10±9*
Depression Diagnosis	0	2	0	0
Insp. time (ms)	665±165	594±164	733±160	671±91
Head injuries	2	3	3	1
Lifetime alcohol (total drinks)	1,552±3,008	5,117±6,312*	6,418±5,633*	2,838±4,761
Lifetime tobacco (total cigarettes)	1,918±9,010	14,355±18,192* §	45,368±46,411* §	3,721±7,693*

Data are mean±standard deviation.

* Significantly different from control group (P<0.05).

§ Significant difference between cannabis group and the ecstasy or methamphetamine groups (P<0.05).

### Drug History

Fifteen subjects had consumed prescribed medication in the month prior to the experiment, but only 2 medications had actions on the central nervous system. One subject had consumed steroid and testosterone medications (up until 15 days prior to the experiment) and another subject had consumed a nicotinic receptor partial agonist (varenicline tartrate, i.e. Champix®) during the 3 days prior to the experiment (to aid cessation of smoking). Consumption of over-the-counter medications included pain relief medications (n = 16 subjects, consumed 1–21 days prior to experiment), antihistamines and other decongestants (n = 6, 0–20 days), heartburn medication (n = 2, 10–21 days), and flu vaccination (n = 1, 7 days). Consumption of complementary medications included vitamin and mineral preparations (n = 16), fish oil (n = 3), herbal preparations (n = 3), and a creatine product (n = 1).

Use of alcohol and tobacco was significantly different between the groups (alcohol: F_4,40_ = 6.073; P = 0.002, tobacco: F_3,47_ = 22.994; P<0.001; Table 1). Lifetime use of tobacco (estimated total cigarettes) was significantly greater in the groups using drugs than in the control group (P<0.001). Lifetime tobacco use was also significantly greater in the ecstasy and methamphetamine groups than in the cannabis group (P<0.02). Eighteen subjects were current smokers (2 control, 6 ecstasy, 4 amphetamine, 6 cannabis) and 4 subjects had consumed tobacco less than 1 hour before arriving at the laboratory. Lifetime use of alcohol (estimated total drinks) was also significantly greater in the ecstasy (P = 0.017) and amphetamine (P = 0.002) groups than in the control group, but lifetime alcohol use did not significantly differ between drug using groups. Subjects had not consumed alcohol in the 12 hours prior to the experiment.

Table 2 shows the percentage of subjects within each group that had used various classes of illicit drugs. Ecstasy was the most commonly used stimulant followed by methamphetamine, cocaine, and recreational use of pharmaceutical stimulants. As expected, poly-drug use was common in the ecstasy and amphetamine groups. All subjects in the ecstasy and amphetamine groups had used cannabis and use of other classes of illicit drugs was also more prevalent in the ecstasy and amphetamine groups than in the cannabis group. The most commonly used hallucinogens were lysergic acid diethylamide (i.e. ‘LSD’) and ‘magic’ mushrooms and the most commonly used inhalant was nitrous oxide.

**Table 2:** Classes of illicit drugs consumed in the ecstasy, amphetamine, and cannabis groups.

	Ecstasy	Amphetamine	Cannabis
Stimulants	100%	100%	0%
Ecstasy	100%	86%	0%
Methamphetamine	78%	86%	0%
Cocaine	44%	43%	0%
Pharmaceutical	22%	14%	0%
Cannabis	100%	100%	100%
Hallucinogens	100%	71%	25%
Opiates	22%	57%	0%
Inhalants	56%	43%	25%
Sedatives	0%	14%	8%
Overdoses (occasions)	0	3	1 (alcohol)

Data are percentage of subjects that have consumed the class of illicit drug in their lifetime. The term ‘hallucinogen’ describes LSD (lysergic acid diethylamide), LSA (d-lysergic acid amide), ‘magic’ mushrooms, DOI (2,5-dimethoxy-4-iodoamphetamine), and/or mescaline. The term ‘opiate’ describes heroin, methadone, opium, and recreational use of codeine, oxycodeine, and/or morphine (total use <3 occasions per subject). The term ‘inhalant’ describes amyl nitrate, ethyl chloride, and/or nitrous oxide. The term ‘sedative’ describes recreational use of benzodiazepine.

Table 3 shows single subject data for lifetime use of ecstasy, amphetamine-like stimulants, and cannabis in the drug use groups. Lifetime use of stimulants (total number of occasions) was significantly greater in the amphetamine group than in the ecstasy group (P = 0.05), but lifetime use of cannabis did not significantly differ between the ecstasy, amphetamine, and cannabis groups. The average duration of abstinence from stimulants was also significantly longer in the amphetamine group (5.5±6.5 yrs; range: 7 days-15 yrs; median: 2 yrs) than in the ecstasy group (0.3±0.3 yrs; range: 11 days-1 yr; median: 61 days; P = 0.028). The average duration of abstinence from cannabis was 0.2±0.3 yrs for the ecstasy group (range: 1 day-0.9 yrs; median: 7 days), 1.1±2.2 yrs for the amphetamine group (range: 1 day-6 yrs; median: 60 days), and 1.5±3.6 yrs for the cannabis group (range: 1 day-12 yrs; median: 26 days). Duration of abstinence from cannabis did not significantly differ between the drug-using groups.

**Table 3:** Summary of lifetime use of cannabis and stimulants in the ecstasy (E), amphetamine (A), and cannabis (C) groups.

		Stimulants		
Subject	Cannabis	Ecstasy	Amphetamine	Total
E1	1529	153	3	156
E2	4380	52	5	57
E3	1456	20	2	22
E4	15	18	1	19
E5	2763	13	4	17
E6	72	9	3	12
E7	183	6	1	7
E8	60	5	1	6
E9	450	5	1	6
E mean (SD)	1212 (1512)	31 (48)	2 (2)	34 (49)
A1	13	1	832	833
A2	7365	3	244	247
A3	6570	1	208	209
A4	360	4	231	235
A5	212	5	92	97
A6	270	1	26	27
A7	4384	0	7	7
A mean (SD)	2739 (3275)	2 (2)	234 (281)	236 (281)
C1	8395			
C2	1248			
C3	364			
C4	154			
C5	130			
C6	104			
C7	92			
C8	80			
C9	64			
C10	39			
C11	9			
C12	6			
C mean (SD)	890 (2388)			

Single subject data are presented (number of times used) and the mean±standard deviation for each group. The term ‘amphetamine’ describes amphetamine and amphetamine-like drugs such methamphetamine, cocaine, dexamphetamine, and methylphenidate. The term ‘ecstasy’ describes ecstasy and MDA (3,4-methylenedioxyamphetamine, 2 subjects).

### Tremor During Movement

Figure 1 shows a raw acceleration trace from one subject during self-paced movement (E) and the result of the fast Fourier transform (F). There was a large peak in the fast Fourier transfer in the physiological frequency range.

**Figure 2:**
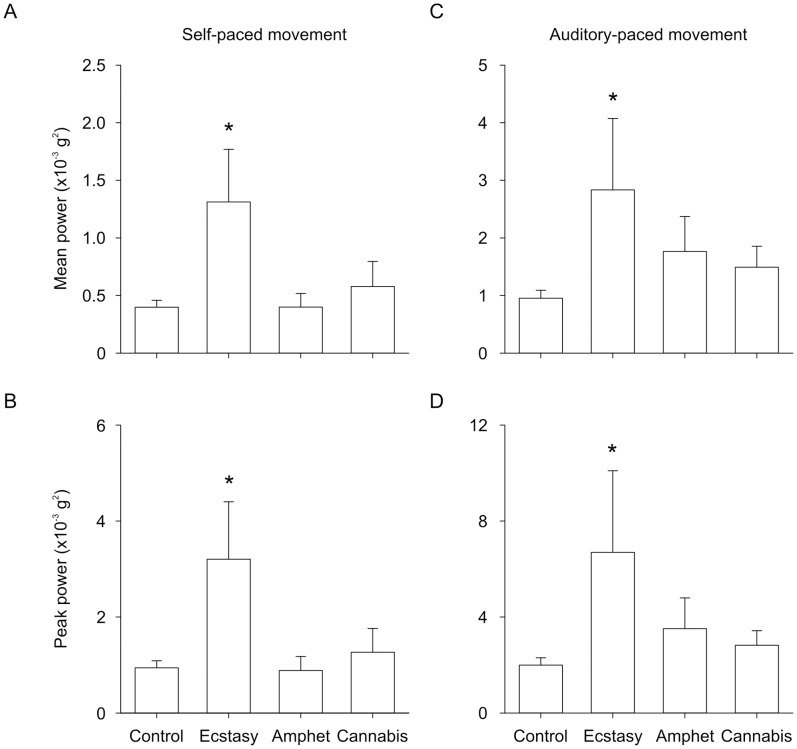
Group data showing the amplitude of tremor in the physiological frequency range (7.4–13.3 Hz). The amplitude of tremor (i.e. power in the fast Fourier transform) for the ecstasy, amphetamine, cannabis, and control groups are shown. A) Mean power in self-paced movement. B) Peak power in self-paced movement. C) Mean power in auditory-paced movement. D) Peak power in auditory-paced movement. *Significantly different from control group (P<0.017).

Figure 2 shows group data for tremor amplitude (i.e. power) in the physiological frequency range (7.4–13.3 Hz) during self-paced and auditory paced movement. The amplitude of tremor in the physiological frequency range differed between groups and between self- and auditory-paced movement. There was a significant main effect of group on the mean (F_3,47_ = 2.839; P = 0.048; Fig. 2A and C) and peak (F_3,47_ = 2.841; P = 0.049; Fig. 2B and D) amplitude of tremor. Tremor amplitude was significantly larger in the ecstasy group than in the control group (P<0.017) but tremor amplitude in the amphetamine and cannabis groups did not significantly differ from control. There was also a significant main effect of movement condition on mean (F_1,47_ = 31.763; P<0.001) and peak (F_1,47_ = 18.768; P<0.001) amplitude of tremor. Tremor amplitude was significantly larger in the auditory-paced movement (Fig. 2C and D) than in the self-paced movement (P<0.001; Fig. 2A and B). There was no significant main effect of group or condition on the peak frequency of tremor and there was no significant interaction (data not shown). There was no correlation between drug history parameters and tremor in the physiological frequency range.

**Figure 3:**
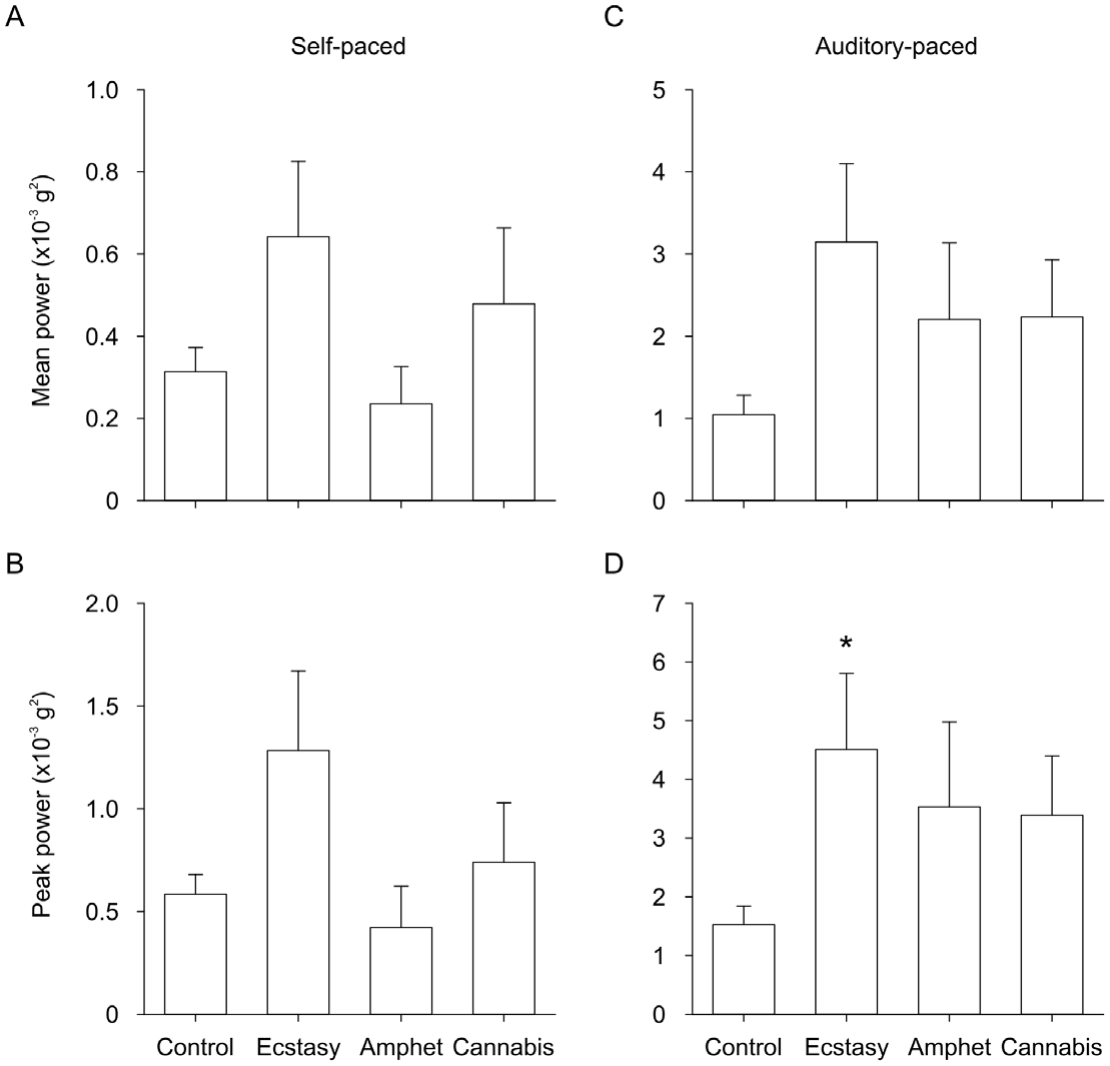
Group data showing the amplitude of tremor in the pathological frequency range (3.9–6.6 Hz). The amplitude of tremor (i.e. power in the fast Fourier transform) for the ecstasy, amphetamine, cannabis, and control groups are shown. A) Mean power in self-paced movement. B) Peak power in self-paced movement. C) Mean power in auditory-paced movement. D) Peak power in auditory-paced movement. *Significantly different from control group (P = 0.034).

Figure 3 shows group data for tremor amplitude (i.e. power) in the pathological frequency range (3.9–6.6 Hz) during self-paced and auditory paced movement. Tremor amplitude in the pathological frequency range differed between self- and auditory-paced movement. There was a significant main effect of movement condition on the mean (F_1,47_ = 41.584; P<0.001) and peak (F_1,47_ = 46.040; P<0.001) amplitude of tremor. Tremor amplitude was significantly larger in the auditory-paced movement (Fig. 3C and D) than in self-paced movement (P<0.001; Fig. 3A and B). There was a trend for a main effect of group on the mean (F_3,47_ = 2.533; P = 0.068) and peak (F_3,47_ = 2.642; P = 0.060) amplitude of tremor and a significant group-by-condition interaction (peak: F_3,47_ = 3.125; P = 0.035; mean: F_3,47_ = 2.689; P = 0.057). Post-hoc analysis revealed that the peak amplitude of tremor was significantly larger in the ecstasy group than in the control group in auditory-paced movement (P = 0.034; Fig. 3D), but the effect did not reach statistical significance in self-paced movement (Fig. 3B). There was no significant main effect of group or group-by-condition interaction on the peak frequency of tremor. There was no correlation between drug history parameters and tremor in the pathological frequency range.

### Tremor at Rest

The mean and peak amplitude of tremor in the physiological (7.4–13.3 Hz) and pathological (3.9–6.6 Hz) frequency range did not significantly differ between groups.

## Discussion

The aim of the study was to investigate the effect of illicit stimulant use on tremor in healthy adults. The results suggest that abstinent ecstasy users exhibit an abnormally large tremor during movement. However, individuals with a history of use of amphetamine-like stimulants and cannabis exhibit normal tremor.

### Tremor During Movement

In both types of movement, tremor was significantly larger in abstinent ecstasy users, but not in individuals that have previously used amphetamine-like stimulants and/or cannabis. The abnormally large tremor was observed in the physiological frequency range (7.4–13.3 Hz) and was robust given that statistical significance was observed for both peak and mean power. The abnormal tremor was also observed at frequencies lower (3.9–6.6 Hz) than the physiological range but statistical significance was only achieved in this range for auditory-paced movement. Data in the 0–3.9 Hz range was not investigated because voluntary movement occurred within this frequency range.

The abnormal tremor in the ecstasy group was surprising given that the majority of individuals in the group had minimal to moderate lifetime ecstasy use. Seven of the 9 subjects had used ecstasy on less than 20 occasions and 4 subjects had used ecstasy on 5–9 occasions (Table 3). Furthermore, subjects had been abstinent for an average of 3 months.

The mechanism that underlies abnormal tremor in abstinent ecstasy users is difficult to identify in humans. The result of our study suggests that the effect is not associated with the acute mechanism of action of ecstasy because all subjects had a negative urine screen for ecstasy and other drugs such as cocaine, amphetamine/methamphetamine, opiates, and benzodiazepines. The abnormal tremor in the ecstasy group was also not associated with subject characteristics such as age, height, weight, handedness, years of education, and speed of information processing because these parameters were well matched between groups. Neuropsychological performance was normal for individuals in the ecstasy group and symptoms of depression did not significantly differ between the ecstasy, amphetamine, and cannabis groups.

Evidence that suggests that ecstasy could play a specific role in abnormal tremor during movement comes from closer inspection of the amphetamine group. The amphetamine group consisted of individuals with a high lifetime use of amphetamine, and total stimulant use was significantly greater in the amphetamine group than in the ecstasy group (Table 3). Thus, any long lasting effect of amphetamine-like stimulants on tremor should have been apparent in this cohort. Any long lasting effect of alcohol and tobacco should also have been apparent in the amphetamine group because subjects in the amphetamine group tended to consume more alcohol and tobacco than subjects in the ecstasy group (Table 1). However, a factor that could have contributed to the absence of abnormal tremor in the amphetamine group is a longer duration of abstinence in the amphetamine group (5.5±6.5 yrs) than in the ecstasy group (0.3±0.3 yrs). However, both groups did include individuals with recent use.

Abnormal tremor in ecstasy users but not in individuals that have used amphetamine-like stimulants is surprising given that the mechanism of action of amphetamine, methamphetamine, cocaine, and therapeutic stimulants such as methylphenidate (e.g. Ritalin®) are more likely to alter brain regions involved in movement. These drugs cause acute, excess accumulation of primarily dopamine by disrupting synaptic vesicles, inhibiting monoamine oxidase, and/or blocking or reversing vesicular monoamine transporters and dopamine reuptake transporters [32], [33], [34], [35]. However, few studies have examined the association between use of these drugs and movement, although new cases of dystonia and tic disorders have been attributed to cocaine use [11] and choreiform syndrome has been associated with amphetamine use [12], [13]. Abstinent methamphetamine users also exhibit poorer motor performance on timed gait and grooved pegboard tasks [8], [9] and epidemiological data suggests an increased risk (hazard ratio = 2.65) of developing Parkinson’s disease later in life [36].

### How Might Ecstasy Affect Tremor During Movement?

Tremor during movement is thought to arise primarily from pulsatile control of agonist and antagonist muscles [37] and central oscillations in brain activity [38]. However, motor unit discharge properties and activity in muscle stretch reflex pathways also contribute to the peak observed in the physiological range [31].

Animal studies show that MDMA, the main psychoactive ingredient of ecstasy tablets, affects brain structures that are involved in tremor and movement. MDMA binds to pre-synaptic monoamine reuptake transporters causing acute accumulation of 5-HT and noradrenaline, and to a lesser extent dopamine [39]. Excessive accumulation of 5-HT and/or peripheral formation of toxic MDMA metabolites leads to neurotoxicity in serotonergic nerve terminals [40], [41], [42]. Other long-term effects include depletion of 5-HT and tryptophan hydroxylase (rate limiting enzyme in 5-HT synthesis), and decreased SERT density in rats [43], [44], [45], non-human primates [46], and humans [39], [47], [48]. Long-lasting serotonergic dysfunction has been observed in the basal ganglia (striatum), thalamus, and cerebral cortex [49], [50]. These brain regions play an important role in tremor and movement and exhibit central oscillatory activity [51], [52]. The amplitude of central oscillatory activity could be increased in abstinent ecstasy users. This theory is supported by the results of an electroencephalography (EEG) study in humans that showed a global increase in the alpha rhythm (8–12 Hz) that is positively correlated with increasing extent of ecstasy use [53]. Furthermore, use of electromagnetic tomography has demonstrated acute changes in the spatial distribution of electrical activity within the brain following a single dose of ecstasy [54].

There are a small number of case studies that also suggest a possible link between ecstasy use and tremor. Ecstasy use has been associated with acute dystonic reaction in the neck and coarse action tremor in the upper limb ([55],c.f. [56]) and postural tremor 10 days after ecstasy intoxication [57]. Furthermore, use of selective serotonin reuptake inhibitors (e.g. fluoxetine), serotonin and norepinephrine reuptake inhibitors (e.g. venlafaxine), and tricyclic antidepressants (e.g. amitriptyline) is associated with abnormal tremor in patients undergoing treatment for depression [58], [59].

It is not known if the abnormal tremor observed in the ecstasy group will persist or improve over time. Recovery of serotonin reuptake transporters has been observed in humans after 12 months of abstinence [60], [61] and axonal sprouting of 5-HT neurons (i.e. re-innervation) has also been observed in rats and non-human primates after 12–18 months of MDMA abstinence [62]. However, abnormal re-innervation patterns occur in non-human primates suggesting that recovery of serotonergic neurons in human ecstasy users may be limited [62].

It is also difficult to investigate the relation between ecstasy dose and the amplitude of tremor during movement. The difficulty arises from variability in the amount of MDMA present in ecstasy tablets (0–250 mg) and the potential presence of other compounds that may affect tremor. In Adelaide, Australia, where the current study was performed, recent chemical analysis of ecstasy tablets suggests that MDMA is the major constituent in 85% of tablets (25–75 mg per tablet) and 50% of tablets contain 100% MDMA [63]. Hence, it is likely that a significant proportion of ecstasy users in the current study were exposed to effective, and possibly neurotoxic, doses of MDMA. Regardless of the actual content of the ecstasy tablets, it appears that individuals who have consumed ecstasy tablets in Australia exhibit an abnormally large tremor during movement.

A limitation of the current study was that postural tremor was not investigated. Assessment of postural tremor is routinely performed during neurological examinations and objective measurement of postural tremor in abstinent ecstasy users is required to provide further clinical relevance for the current findings. An additional limitation is that it is unknown if subjects in the ecstasy group had a pre-existing tremor prior to the onset of illicit drug use.

### Tremor During Relaxation

Resting tremor was unaltered in the ecstasy, amphetamine, and cannabis groups both in the physiological frequency range and at lower frequencies. The result of our study contradicts the findings of an earlier study conducted on abstinent cocaine dependent individuals. Abstinent cocaine-dependent individuals are reported to exhibit an increased resting tremor that occurs at an abnormally low frequency [3]. The contradictory findings are likely due to differences in spectral analysis methodology and uncertainty regarding total stimulant use or use of other types of illicit drugs in the Bauer (1993) study.

In summary, our results suggest that individuals with a history of ecstasy use exhibit an abnormally large tremor during movement that may persist for months after cessation of use. In light of the increasing popularity of ecstasy, further investigation of the long-term neurotoxic and functional consequences of ecstasy use is warranted. Future studies are also required to determine if abnormal tremor during movement translates to increased risk of movement disorders in this population.
